# A Persistent Misconception About Hip Rotation Torques During Elastic Band Sidestepping

**DOI:** 10.3390/jfmk11030266

**Published:** 2026-07-06

**Authors:** Heiliane de Brito Fontana, Walter Herzog, Felipe Neumann, Heron Baptista de Oliveira Medeiros, Marcio Nunes, Vitor Guarda Munari, Josiel Gomes Ribeiro

**Affiliations:** 1 BSiM-UFSC, Musculoskeletal Biomechanics Research Group, Federal University of Santa Catarina (UFSC), Florianópolis 88040-900, Brazil; heiliane.fontana@ufsc.br (H.d.B.F.); neumann.nf@gmail.com (F.N.); heronbomed@gmail.com (H.B.d.O.M.); marcio.nunes@unifebe.edu.br (M.N.); vitorgm.fisioterapia@gmail.com (V.G.M.); josiel.ribeiro@posgrad.ufsc.br (J.G.R.); 2 Human Performance Laboratory, Faculty of Kinesiology, University of Calgary, Calgary, AB T2N 1N4, Canada

**Keywords:** hip kinetics, elastic bands, sidestepping, lateral band walk, transverse-plane torque, electromyography, exercise prescription

## Abstract

Resisted sidestepping is widely implemented in rehabilitation and strength training, and exercise prescription is often guided by recommendations based on surface electromyography (EMG) patterns and intuitive assumptions about how elastic-band placement and posture influence hip loading. EMG provides valuable insight into neuromuscular strategies, but it does not, by itself, specify the direction or magnitude of joint-level mechanical demand. In this opinion article, we argue that exercise prescription is strengthened when EMG findings are interpreted within a joint-kinetic framework, in which the net external joint moment, calculated via inverse dynamics, defines the mechanical demand imposed by the task. Using resisted sidestepping as the central example and drawing on previously published three-dimensional inverse-dynamics findings, we address a common misconception that placing an elastic band around the forefeet necessarily imposes an external hip moment toward medial rotation that can help target “hip lateral rotator” muscles. Available inverse-dynamics evidence indicates that, under typical execution with slight hip and knee flexion, forefoot-band sidestepping imposes a resultant external hip moment toward lateral rotation, thereby requiring a net internal muscular moment toward medial rotation to maintain alignment and perform the task. We further highlight that posture and resistance configuration modulate how demand is distributed across joint movement planes. Specifically, band placement alters the moment arms of the elastic resistance relative to different hip joint axes and therefore influences how changes in band stiffness are translated into transverse- and frontal-plane hip loading. Thus, band placement, posture, and resistance magnitude should be selected according to the intended joint-level loading objective rather than inferred from EMG patterns alone. Although illustrated with sidestepping, this reasoning is relevant to many resistance and rehabilitation exercises in which EMG-only interpretations, without consideration of external forces and joint kinetics, may lead to incomplete or incorrect inferences about joint loading and musculoskeletal function.

## 1. Introduction

Elastic resistance exercises are widely used in rehabilitation and strength training to develop function in weight-bearing tasks. Specifically, the resisted sidestepping exercise is commonly used to target the “hip abductors” and “lateral rotators” [1,2,3,4,5]. In a recent review, Gonzáles-de-la-Flor (2025) [1] provides a timely synthesis for clinicians seeking practical guidance to prescribe sidestepping and monster walk exercises. The concluding recommendation presented by the author for strengthening the target muscles—distal band placement (ankles or forefeet) combined with a mini-squat posture (≈20–30° hip and knee flexion)—can be clinically appealing and is consistent with common practice. This recommendation is based primarily on previous studies that investigated electromyographic (EMG) activation of hip muscles during sidestepping exercises with the band placed at different locations on the lower limb [3,4,5].

The biomechanical rationale used to justify this recommendation is commonly grounded in two assumptions that frequently appear, explicitly or implicitly, in clinical interpretations of weight-bearing exercises: (i) that surface electromyography (EMG) can be used to infer muscular demand in the targeted joint, and (ii) that distal band placement around the forefeet imposes an external hip torque demand toward adduction and medial rotation. While EMG provides valuable insight into neuromuscular activation patterns and is widely used to inform exercise selection, its interpretation in terms of joint-level mechanical demand is not always straightforward—particularly for hip muscles, which are known for their complex architectures and their posture-dependent moment arms. In such contexts, the first assumption warrants further examination.

Importantly, the latter assumption is contradicted by experimental evidence that directly quantified three-dimensional hip torques during sidestepping [2]. In this opinion article, we argue for a mechanics-first framing of exercise prescription: the net external joint moment (as estimated via inverse dynamics when available, or through mechanically coherent reasoning about forces relative to joint axes) should define joint-level demand, with EMG interpreted as part of the neuromuscular response to that demand. Our goal is not to dispute the value of sidestepping in rehabilitation or the use of EMG, but to clarify joint-level mechanics so that band placement and posture can be chosen to match the intended training stimulus.

Throughout this opinion article, we use **external torque** to denote the **net external joint torque demand** (inverse-dynamics resultant). **Internal torque** refers to the net muscular torque required to counter that demand. Data used to support this opinion article were previously published [2].

## 2. The Sidestepping Exercise

Movement-based (“functional”) exercises with elastic bands are increasingly prescribed, and the demand can be adjusted through several parameters, among which the stiffness of the band, the location of the band, as well as the posture adopted during the exercise, are particularly useful. Sidestepping consists of cyclic lateral stepping with elastic resistance, initiated by lateral advancement of the non-support limb during single-limb support, followed by a period of widened double support for load transfer, a controlled trailing of the posterior limb against band recoil, and a brief narrow double-support phase preceding the subsequent step [2,5]. There has been great interest in this exercise in the last decade, which is reflected in the number of studies aimed at characterizing the demand of this exercise under different band placement and kinematic conditions [1].

Distal band placement around the forefeet during sidestepping has frequently been justified by EMG evidence suggesting greater gluteal myoelectric activity and a reduced relative contribution of the tensor fasciae latae (TFL) [1,3,4]. Specifically, moving the band distally (knees → ankles → feet) is generally associated with increased gluteus maximus and gluteus medius activation, whereas TFL activity tends to increase from knees to ankles but does not consistently increase further when the band is moved from the ankles to the feet [3,4]. The myoelectric activity of hip muscles will also depend on methodological differences that can influence normalized EMG and between-study comparability, including the exercise variant tested, participant characteristics (e.g., sex), whether outcomes reflect mean vs. peak EMG, how phases/limbs are defined, and how posture is executed and quantified [3,4,5,6]. The observations of an increased activation of gluteal muscles relative to the TFL have encouraged the interpretation that distal placement preferentially “targets” hip lateral rotators (attributed to the gluteal musculature) while minimizing hip medial rotators (attributed to the TFL). Yet, because EMG reflects neuromuscular strategy rather than joint-level mechanical demand, these activation patterns do not establish the direction of the net external hip rotation moment during the task—a point that is central to the persistent misconception addressed here [1,7].

A commonly proposed interpretation is that distal band placement introduces an external torque that tends to “bring the toes in,” thereby promoting hip medial rotation and contributing to an increased gluteal-to-TFL EMG ratio. In the review by González-de-la-Flor [1], this interpretation is articulated by suggesting that “forefoot placement imposes additional external torque demand toward hip medial rotation, which selectively enhances gluteus maximus activation while minimizing the recruitment of the TFL.” Additionally, EMG studies have also shown that a squat posture increases gluteal EMG amplitudes and reduces TFL EMG amplitudes compared with a more upright posture [5]. While this reasoning is consistent with observed EMG patterns, the associated assumptions behind these recommendations warrant closer scrutiny.

## 3. Hip Muscle Function and the Limits of EMG

EMG provides valuable information about neuromuscular strategies. In addition to commonly reported amplitude-based outcomes, EMG can be analyzed using temporal parameters, including activation onset/offset, contraction sequence, and coordination patterns, as well as frequency-domain features related to neuromuscular control or fatigue. These analyses can help characterize the organization of muscle activity during movement and can be useful for assessing how muscles are recruited across phases of an exercise.

However, EMG-derived variables—whether amplitude-, time-, or frequency-based—do not, by themselves, determine the direction or magnitude of the net external joint moment imposed by a task. Nor do they satisfactorily resolve how the required net joint torque is distributed across individual muscles, a problem commonly referred to as “force sharing”. Inferring individual muscle forces from surface EMG alone requires assumptions about muscle lines of action, muscle properties, and coordination strategies that are rarely satisfied in vivo. In fact, the EMG–force relationship is context dependent [6] and can be influenced by muscle length, contraction velocity, tendon compliance, architecture, and history-dependent effects, posing significant challenges to the determination of muscle force from EMG [6,7,8,9,10,11,12].

The limitations of surface EMG as a proxy for mechanical demands and the complexity of neuromuscular control are not limited to the ones described above and have been discussed across exercise physiology, sport science, and simulations [8,9,13,14]. With regards to the rationale used to justify distal band placement during sidestepping, the interpretative gap between EMG activity and the mechanical loading of the hip is particularly important due to the complexity of the hip joint musculature and the risk of misjudging the direction of the torque produced by hip muscles. Erroneous, or at least oversimplified, muscle actions around the hip transverse and frontal planes are often used to interpret hip myoelectric activities during functional exercises.

Moment arms of hip muscles can change dramatically with hip posture [15,16], and the architecture of large muscles, such as the gluteus maximus and medius, allows for different muscle compartments/fascicles to have opposite muscle actions within a hip axis [17,18]. Simplistic labels based on the anatomical position (e.g., “medial” or “lateral” rotator) are therefore often misleading and may not generalize to the joint positions used during functional exercise. For example, although it is sometimes claimed that there is a “consensus” that the TFL contributes to hip internal rotation [19], experimental and cadaveric modeling evidence suggest that the TFL has a negligible medial-rotation moment arm across relevant hip positions [15,20]. Consistent with this, we previously showed that TFL activation can increase systematically when an external medial-rotation torque demand is controllably imposed at the hip [17]. Accordingly, elevated TFL activation during functional exercises may reflect stabilization demands, co-contraction, and multi-planar task requirements (including roles in the frontal and sagittal planes) that are not captured by a single-action label. Similar complexity applies to the gluteal musculature: depending on hip flexion angle and the compartment considered, portions of gluteus maximus and gluteus medius may contribute to either medial or lateral rotation; notably, with hip flexion as in a squatted posture, large regions of both muscles can exhibit moment arms toward medial—not lateral—rotation [15,16,19,20]. Collectively, these features reinforce the need to interpret hip EMG within a mechanically defined loading context, in which the direction and magnitude of the net external joint moment are established before drawing conclusions about “targeted” rotator demand.

## 4. Joint Kinetics and the Interpretation of Functional Exercise Demands: What the Mechanics (Hip Torque Data) Show and Why Intuition Fails

A practical way to connect joint mechanics and muscle function in exercise prescription is to treat external loading as the primary constraint: the net external joint moment defines the minimum agonistic internal muscular moment required to perform the movement, whereas EMG-derived recruitment patterns reflect the myoelectric activity associated with one of many feasible neuromuscular solutions shaped by muscle–tendon mechanics, coordination goals, and co-contraction. From this perspective, a central question in many strengthening contexts is whether an exercise imposes a joint-level demand with the intended direction and sufficient magnitude. At the same time, there are clinical scenarios where preferential recruitment of a specific muscle or region is desirable; in those cases, establishing the direction of the external joint moment helps ensure that activation-based progressions are not built on an incorrect assumption about the underlying loading.

Interpretive errors about resisted sidestepping may arise when band placement, visible limb motion, and EMG patterns are interpreted as direct indicators of hip rotation loading, while kinetic evidence is absent from the discussion. Because inverse-dynamics analyses are more demanding to implement than EMG recordings, the available literature is often weighted toward EMG outcomes, which can encourage mechanistic inferences and exercise recommendations [1,3,4,7] that are not explicitly verified or supported by experimental evidence [2].

**Figure 1:**
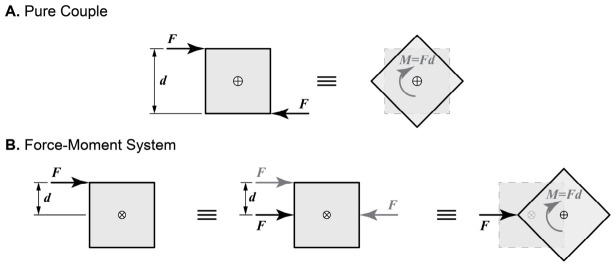
(**A**) Pure couple (free moment). Two equal, opposite, and parallel forces separated by a perpendicular distance d generate a net moment (Μ=F·d) with zero resultant force. The effect is a pure rotational tendency, with no net translational effect on the center of mass. (**B**) Force–moment system. A single force F applied with a nonzero moment arm relative to the center of mass produces a combined loading consisting of a nonzero resultant force and an associated moment (Μ=r×F), with magnitude F·d for a perpendicular offset d. This system is mechanically equivalent to the same force applied at the center of mass, together with an added free moment about the center of mass. Unlike a free moment, the moment produced by a resultant force results in both translational and rotational effects; therefore, the two loading conditions lead to different kinematics, with the free moment’s effect being independent of its location, while the moment produced by a force depends on the location of the force relative to the target axis.

Mechanically, the misconception around sidestepping hip torques can be traced to conflating two distinct constructs: (i) a free moment (a pure torque applied without a net force), and (ii) a moment generated by a resultant force acting with a moment arm. In Figure 1, the difference between these two loading conditions is illustrated.

In sidestepping, the commonly assumed advantage of placing an elastic band around the feet—rather than at more proximal segments (e.g., the thigh)—for strengthening the hip lateral rotators is often based on the idea that a moment is generated at the feet that promotes medial displacement of the toes and a medial rotation of the lower limb about the hip. This interpretation implicitly treats the loading as equivalent to a free moment (A). However, the band generates a force–moment system (B), not a free moment.

The band force tends to draw the feet toward the midline (medial translation), while simultaneously creating a moment about the hip, the direction of which depends on the moment arm of the band force around the hip. The band-force vector is directed toward the contralateral limb, and under typical execution, its line of action passes posterior to the longitudinal axis of the thigh (schematically shown in Figure 4A), generating an external hip moment toward lateral rotation [2]. Consequently, maintaining limb alignment requires an internal muscular torque toward medial rotation. However, the hip moment generated by the band is not the sole determinant of the net intersegmental hip torque, as the resultant joint torque also depends on ground reaction forces, segmental inertia, and movement execution. This distinction is developed further below and is particularly important in weight-bearing exercises, where band-generated moments and resultant joint torques are often conflated.

Inverse dynamics can be used for estimating net intersegmental (resultant) joint torques, i.e., the net external joint torque demand implied by kinematics, inertial effects, and measured external forces, allowing the mechanical demand of an exercise to be calculated. From a training and rehabilitation perspective, one should recognize that for an exercise to selectively challenge a muscle group, an external resistance must oppose the action of that group at the joint. In simple, non-weight-bearing tasks, such as a biceps curl with a dumbbell, this relationship can be intuitive, and inferences based on EMG are often reasonable as long as the direction and magnitude of the external load are obvious.

**Figure 2:**
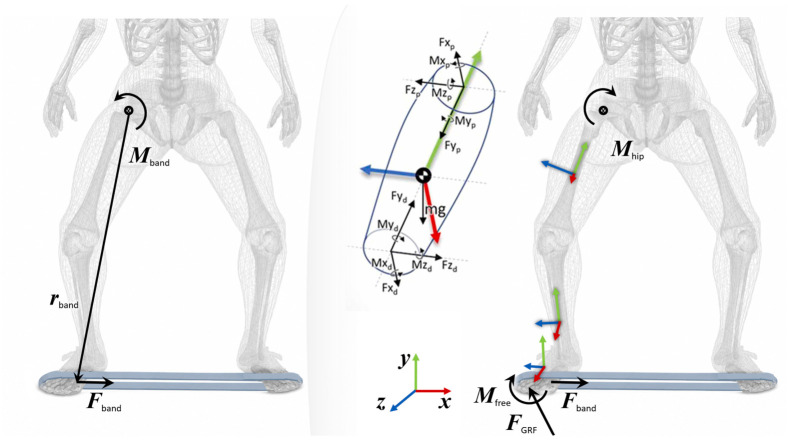
(**Left**) During sidestepping with an elastic band around the forefeet, the band applies a tensile force to each foot. This force is directed approximately along the line of the band, toward the opposite foot. The mechanical effect of this force on the hip is determined not only by the magnitude of the band force, but also by where the force is applied relative to the hip joint center. In mechanics, the external moment generated by the band about the hip is given by the cross product between the position vector from the hip joint center to the point of force application and the band force vector: ${Μ}_{band}=r_{band}\times F_{band}$ where $r_{band}$ is the vector from the hip joint center to the point where the band force acts on the foot, and $F_{band}$ is the force exerted by the band on the foot. The direction of the resulting torque is perpendicular to the plane defined by these two vectors and follows the right-hand rule. (**Right**) The net external torque acting about the hip during sidestepping is not determined solely by the elastic band. Rather, it emerges from the combined action of all external forces acting on the body segments, each acting through its own moment arm relative to the hip joint center. In inverse dynamics, the net external hip torque is the sum of the torques generated by all external forces acting on the system of interest, including the band force, the ground reaction force, gravitational forces acting on the segment, and—for dynamic conditions—the inertial effects associated with the segment’s linear and angular accelerations. For example, even if the force of the elastic band produces an external torque tending toward hip lateral rotation, the resultant net external hip torque also depends, among other things, on how the ground reaction force vector is oriented relative to the hip joint center. For more details regarding the calculation of hip torques, refer to [2].

In contrast, functional exercises often involve closed-chain phases with periods of double-leg support. Under these conditions, joint-level demands are not readily inferred from posture, band position, or EMG patterns alone. During the double support phase, the system is mechanically indeterminate: the same kinematics and band elongation can be achieved with infinitely many combinations of ground reaction force magnitudes and directions. Consequently, net joint torques and the muscular strategies required to produce them cannot be determined from kinematics and band force alone without measuring/estimating GRFs and their distribution (Figure 2).

It is instrumental to distinguish between the moment generated by the elastic band itself and the resultant joint torque determined via inverse dynamics. The moment produced by the force of the band around the hip is a geometric quantity defined by the cross product of the band force and its moment arm relative to the hip joint. The resultant hip joint torque, however, depends on all external moments and forces acting on the leg, including the contribution from the band forces, ground reaction forces, and the leg’s inertia tensor and acceleration. This is particularly relevant for the double support phase of functional exercises with elastic resistance. For example, a participant can, at least theoretically, rely on ground reaction forces to counter the band force (as hypothetically shown in Figure 2, right—note the directions of the GRF), which would produce joint torques that differ substantially from those intuitively inferred from band direction (note the opposite directions of the hip moments illustrated in the left and right panels of Figure 2).

**Figure 3:**
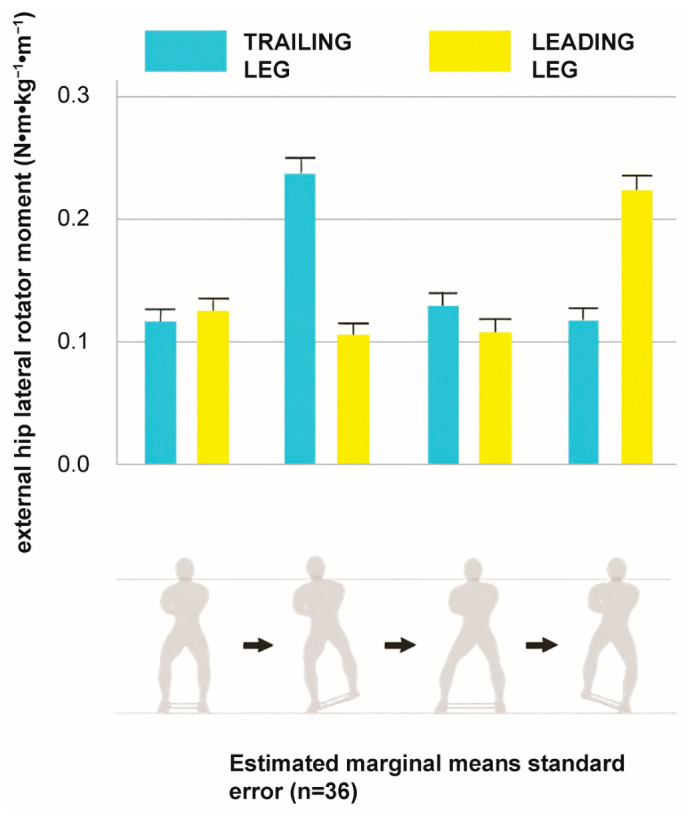
Mean and standard error for the externally imposed hip torque in the transverse plane for the trailing and leading limbs across all phases of the sidestepping task performed with a looped resistance band placed around the forefeet. Since there was no interaction with the direction of stepping, data were pooled across leftward and rightward displacements. Across phases and limbs, the net external hip rotation moment was consistently directed toward **lateral rotation**, requiring a corresponding net **internal** muscular moment toward **medial rotation** to maintain alignment (reprocessed from [2]).

Using inverse dynamics, Medeiros et al. [2] quantified net external hip torques during resisted sidestepping across movement phases and postures. In this experimental analysis of 36 adults performing sidestepping with an elastic band positioned around the forefeet [2], the authors directly tested the assumption—recently reiterated in González-de-la-Flor’s review [1]—that distal band placement externally imposes a hip medial-rotation torque. This assumption was not supported. Instead, the exercise consistently generated a net external hip moment demand toward lateral rotation across all movement phases and in both the leading and trailing limbs [2] (Figure 3). These findings challenge commonly cited recommendations [1,3,4,7] that interpret forefoot band placement as preferentially strengthening the hip lateral rotators through an externally imposed medial-rotation demand.

While inverse dynamics analyses provide robust and accurate mechanical insight, they remain rare in clinical and rehabilitation studies because they are time-consuming technically demanding, and require expensive equipment systems, while EMG-based assessments are comparatively easy to implement, less time consuming, and they are relatively less expensive. This imbalance likely contributes to the continued reliance on EMG-centered interpretations and accompanying fragile assumptions regarding joint moments. Importantly, inverse dynamics approaches offer a principled solution by accurately quantifying resultant intersegmental joint torques. Resultant torques obtained in this manner represent the minimum agonistic muscular torque required to satisfy the movement dynamics but do not resolve individual muscle forces. Antagonistic coactivation, expected in many functional and rehabilitative tasks, is not captured explicitly but is embedded implicitly in the net solution.

It is neither realistic nor necessary for clinicians to calculate joint torques for every exercise they prescribe. Nevertheless, one must appreciate how external resistances alter movement kinetics and recognize when intuitive interpretations may be misleading. In the context of closed-chain, weight-bearing tasks with elastic resistance, joint-level demands cannot be inferred from kinematics or EMG alone, and inverse dynamics, while often limited to laboratory-based analyses, are useful for an understanding of the mechanics of the task.

Recent technological advances offer promising avenues to bridge the gap between laboratory-based inverse-dynamics analyses and assessments in the clinical setting. Musculoskeletal simulations driven by inertial measurement units, as well as open-source platforms that estimate kinematics and kinetics from smartphone video, are rapidly expanding access to quantitative movement analysis. However, these approaches currently face important limitations, especially in the context of elastic resistance exercises. Forces generated by elastic bands are not yet automatically detected or modeled, and closed-chain conditions, particularly double support phases, cannot be resolved confidently without external force measurements. As such, while these newly available tools are promising for field-based individual characterization, they are not yet sufficient to determine joint kinetics in exercises involving elastic resistance.

In closing, correct identification of net external joint torque direction provides a necessary foundation upon which interpretations of muscular demands are built. As emerging technologies continue to lower the barrier to kinetic analysis, integrating joint-level mechanical assessments into exercise prescription will soon be possible in different contexts for aligning clinical intent with biomechanical characteristics of the exercise.

## 5. Posture Matters: Flexion Increases the Externally Imposed Transverse-Plane Demand

**Figure 4:**
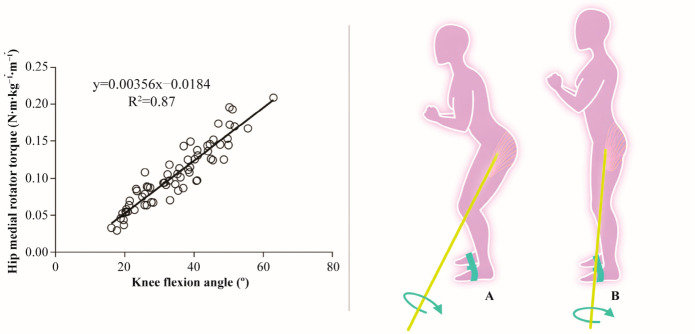
(**Left**) Relationship between knee flexion angle and the magnitude of the net external hip rotation moment in the transverse plane during resisted sidestepping. The regression includes data from 36 participants performing the exercise in two postural conditions—upright and semi-squatted—resulting in two data points per participant. The relationship indicates that knee flexion angle explained 87% of the overall variability in the net external hip rotation moment (R^2^ = 0.87). Larger knee flexion angles were associated with larger externally imposed hip lateral rotation moments (plotted as positive values). (**Right**) Schematic illustrating how posture modulates transverse-plane loading when a looped elastic band is placed around the forefeet. The hip longitudinal axis is illustrated along the thigh. (**A**) In a semi-squat posture, increased hip/knee flexion increases the perpendicular moment arm of the band force about the hip’s longitudinal axis, resulting in a larger externally imposed hip rotation moment for a given band force. (**B**) In a more upright posture, the smaller moment arm results in a smaller externally imposed hip rotation moment for a given band force (reproduced from [2]).

Postural changes during sidestepping influence both neuromuscular strategy [5] and mechanical demand. In our study, participants performed the side-stepping task with a looped elastic band under two prescribed conditions—an upright posture and a semi-squatted posture—to test how hip/knee flexion affects hip joint moments [2]. Across these conditions, greater knee/hip flexion substantially increased hip torques in the transverse and sagittal planes, whereas frontal-plane torques were comparatively less affected [2]. Notably, knee flexion angle alone explained 87% of the variability in the hip rotation moment: increasing knee flexion increased the magnitude of the net external hip moment toward lateral rotation (Ref. [2], Figure 4), thereby increasing the required net internal muscular moment toward medial rotation to maintain limb alignment.

Mechanistically, this result is explained by changes in segment orientation with increasing flexion, which increases the perpendicular moment arm of the band force relative to the hip longitudinal axis, thereby amplifying transverse-plane torque demands for a given band force.

These findings suggest that combining a squatted posture with distal band placement increases transverse-plane hip loading, which could be undesirable when the clinical goal is to keep internal medial-rotation torque demands modest, such as during early rehabilitation or in individuals with low tolerance to rotational loading. This mechanical trade-off is important because EMG-based recommendations often emphasize increased gluteal activation with distal band placement or greater hip/knee flexion [3,4,5,6], whereas EMG alone does not identify the direction or magnitude of the external joint moment that must be countered by the musculoskeletal system. Thus, an exercise configuration that may appear advantageous from an activation-based perspective may impose a joint-level torque demand that differs from the intended mechanical stimulus.

The influence of posture on joint moments has been examined extensively in resistance exercises, particularly in squat-related tasks performed with external loads [18,21,22,23]. These studies show that changes in posture and segment orientation may substantially alter joint moment distribution, supporting the broader principle that exercise technique modifies mechanical demand [18,23,24,25]. However, most of this literature involves relatively simple loading configurations, such as gravity-based external loads, in which the direction and point of force application are comparatively well defined. The few studies in which elastic resistance was included in biomechanical analyses of exercise involving body weight support often did not require the band force to be explicitly incorporated into the inverse-dynamics calculation for the joint of interest [26,27]. For example, if a band is placed around the thighs and the outcome of interest is the knee moment, the band force acts proximal to the knee and therefore does not directly enter the distal free-body diagram used to calculate knee joint moments. In contrast, when elastic resistance is applied within the kinetic chain relevant to the joint being interpreted—such as a band acting at the feet or shanks while hip loading is evaluated—the band force interacts with ground reaction forces, segment orientation, and inertial effects, requiring a model that incorporates these elements. Consequently, the effect of elastic resistance applied within the kinetic chain on joint moments during dynamic, multi-planar tasks remains comparatively understudied, despite its relevance for functional rehabilitation exercises.

From a clinical standpoint, addressing this gap is important to support the deliberate use of posture, band position, and band stiffness as strategies to shape joint loading in accordance with rehabilitation goals. Elastic resistance is widely used to provide direction-specific loading during functional tasks. However, without a clear understanding of how exercise parameters such as posture and band position interact with resistance magnitude to shape joint moments, clinicians may rely primarily on empirical adjustments or on current recommendations that do not necessarily reflect the joint-level mechanical demands imposed by the task. Incorporating mechanically coherent reasoning about band force direction, moment arms, and joint axes may therefore help clinicians select exercise configurations that better match the intended loading objective.

## 6. Practical Implications: Matching Band Placement to the Intended Mechanical Goal

There may be clinically valid reasons to prefer a squatted posture during sidestepping—enhanced stability, an “athletic stance,” or sport-specific similarity. The key issue is that posture and band placement should be selected with awareness of their joint-level consequences.

**Figure 5:**
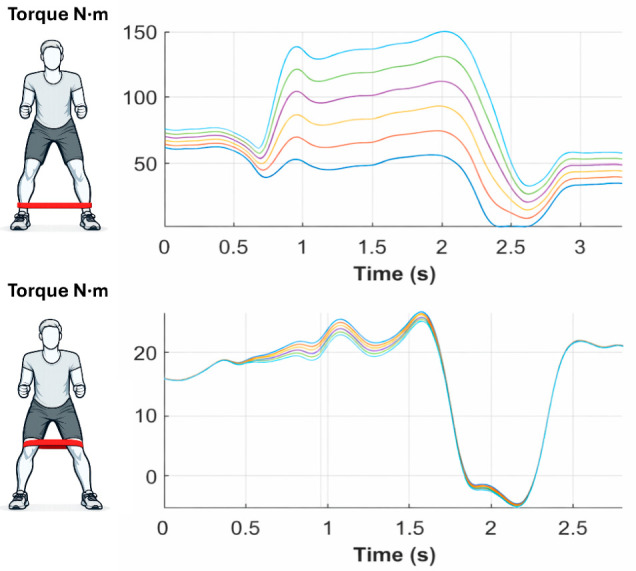
Hip torque curves in the transverse plane (N·m) during the side-walking exercise performed in a semi-squatted position with the elastic band positioned at the shank (**top**) and at the thigh, above the knees (**bottom**). Curves are shown for one representative participant using the same experimental kinematics and inverse-dynamics model while band stiffness was manipulated in the model. Different colors represent different modeled elastic stiffness levels. When the band is placed around the shank, increases in band stiffness markedly increase transverse-plane hip torque magnitude, consistent with a substantial transverse-plane moment arm. In contrast, when the band is placed around the thigh, hip rotation torques remain minimal, and increases in band stiffness result in negligible changes in transverse-plane torque magnitude, consistent with a small/negligible transverse-plane moment arm. The net external hip torque, estimated as the inverse-dynamics resultant, is plotted; positive values indicate external torque toward hip lateral rotation. Because these data are from one representative participant, they are presented to illustrate the expected mechanical behavior rather than to provide group-level estimates (Adapted from material previously presented by the authors at the XVIII Brazilian Congress of Biomechanics, Manaus, Brazil, 2019).

If the intent is to minimize the externally imposed hip moment toward lateral rotation (and consequently reduce the internal demand toward medial rotation), a more proximal band placement (around the thigh, above the knees) may represent a biomechanically sound alternative. While neuromuscular responses may vary across individuals [4], placing the band closer to the hip’s longitudinal axis is expected to reduce the perpendicular moment arm of the band force about that axis, thereby reducing the transverse-plane hip rotation moment. This behavior is illustrated for one representative participant in Figure 5: when the band is placed distally (around the shank/ankles), transverse-plane hip rotation torque increases markedly as band stiffness increases (top panel), consistent with a substantial transverse-plane moment arm. In contrast, when the band is placed proximally (around the thighs), transverse-plane hip rotation torques remain small and show minimal sensitivity to band stiffness (bottom panel), consistent with a small/negligible transverse-plane moment arm. Moving the band proximally is expected to reduce frontal-plane loading because the frontal-plane moment arm is shortened; however, this can be compensated for by increasing band stiffness. Importantly, because the transverse-plane moment arm remains small with thigh placement, increasing stiffness has little effect on hip rotation torque, whereas changes in frontal-plane torque are still expected because a nonzero moment arm remains in that plane.

This point is clinically actionable: clinicians can “tune” the exercise—i.e., adjust the relative magnitude and orientation of joint torque demand—by selecting band placement according to the relative transverse- versus frontal-plane emphasis, and using it to modulate how different band stiffness will affect hip loading across planes.

## 7. Final Considerations and Perspectives

Sidestepping remains a valuable component of lower-limb rehabilitation, and the review by González-de-la-Flor [1] contributes by synthesizing an applied literature for clinicians. Our objective is narrower: to correct a persistent misconception about the direction of the net external hip rotation torque demand during distal-band sidestepping and to emphasize that posture and elastic band placement modulate the magnitude of transverse-plane demand.

EMG studies report that distal placement increases gluteal activation and can “improve” gluteal-to-TFL activation ratios [3,4], and that a mini-squat posture may further increase gluteal EMG while reducing TFL EMG [5]. These findings describe neuromuscular strategies, but they do not define joint-level mechanical demand.

Direct kinetic evidence indicates that forefoot-band sidestepping imposes a net external hip torque demand toward lateral rotation (adduction and flexion) across phases and in both limbs [2], requiring internal hip muscular torque toward medial rotation, abduction, and extension to maintain alignment. Moreover, increased hip/knee flexion amplifies this transverse-plane demand [2]. Accordingly, when feasible, exercise prescription should be informed by joint kinetics (e.g., inverse dynamics) or by mechanically coherent reasoning about external forces relative to joint axes, rather than by EMG patterns or band placement alone.

The arguments presented in this opinion article are primarily grounded in our previously published experimental work investigating joint torques during sidestepping [2]. A key limitation is that inverse dynamics estimates the net intersegmental hip joint moment (resultant internal torque demand), but it does not resolve how that net moment is distributed across individual muscles. In the hip angular range used during sidestepping, passive structure contributions are expected to be small; thus, the net intersegmental hip moment primarily reflects the combined action of the muscles crossing the hip. Nevertheless, this approach does not provide muscle-specific forces or moments (i.e., it cannot determine “force sharing” among agonists and antagonists). Resolving force sharing in vivo remains non-trivial and requires substantial assumptions even with state-of-the-art methods [28]; accordingly, interpretations of muscle-specific loading during functional tasks remain qualitative and can be misleading. Importantly, however, establishing the direction and magnitude of the net external hip moment demand—as provided by inverse dynamics—remains foundational: it defines the required *resultant* internal muscular moment to execute the task and provides the mechanical context within which any discussion of muscle-specific contributions must be interpreted.

Future work should expand the sparse literature quantifying joint torques during multiarticular exercises with elastic resistance across band placements, postures, and execution strategies. In the absence of such kinetic evidence, interpretations often revert to intuitive reasoning based on band location, visible limb motion, and EMG patterns—an approach that can be insufficient and may contribute to mechanically fragile recommendations as discussed in this opinion article. Aligning band placement and posture with the intended mechanical objective may improve the specificity, safety, and interpretability of elastic-resistance exercise prescription, supporting a more mechanically grounded framework for rehabilitation and performance contexts. These considerations should be interpreted as hypothesis-generating, as they are not yet supported by intervention studies.

## Data Availability

No new data were collected or analyzed for this opinion article. The experimental findings discussed are derived from previously published work [2].
